# Supplementary material for: Altered DNA methylation within DNMT3A, AHRR, LTA/TNF loci mediates the effect of smoking on inflammatory bowel disease
Source: Nat Commun. 2024 Jan 18;15:595. doi: 10.1038/s41467-024-44841-y (PMC10796384; doi:10.1038/s41467-024-44841-y)
Supplement: Supplementary file 1 — Supplementary Information [file 41467_2024_44841_MOESM1_ESM.pdf]

Altered DNA methylation within DNMT3A, AHRR, LTA/TNF loci mediates the effect of smoking on inflammatory bowel disease

Supplementary Tables Legends

Table S1. The baseline characteristics of UK Biobank participants.
Table S2. Effect estimates of two-sample MR analysis of smoking behaviors with IBD.
Table S3. MP-PRESSO results of two-sample MR analysis of smoking behaviors with IBD.
Table S4. The demographic characteristics of the IBD-CHARACTER cohort.
Table S5. Colocalization analysis for CD and UC.Definition of inflammatory bowel disease in the UK Biobank.
Table S6. Definition of inflammatory bowel disease in the UK Biobank.

Table S1. The baseline characteristics of UK Biobank participants

Characteristic	Crohn's disease Cases (n=1770)	Ulcerative colitis Cases (n=2889)	Controls (n=479973)
**Age at recruitment, mean (SD)**	56.78 (8.14)	57.60 (7.86)	56.53 (8.09)
**Gender, n (%)**			
Male	797 (45.03)	1487 (51.47)	219322 (45.69)
Female	973 (54.97)	1402 (48.53)	260651 (54.31)
**Smoking status, n (%)**			
Never smoking	788 (44.52)	1281 (44.34)	262248 (54.64)
Former smoking	689 (38.93)	1304 (45.14)	165020 (34.38)
Current smoking	282 (15.93)	286 (9.90)	50249 (10.47)
NA	11 (0.62)	18 (0.62)	2456 (0.51)
**Drinking status, n (%)**			
0-12.5 g/day	377 (21.30)	573 (19.83)	108931 (22.70)
12.5-50 g/day	596 (33.67)	1076 (37.24)	183729 (38.28)
> 50 g/day	108 (6.10)	270 (9.35)	37261 (7.76)
NA	689 (38.93)	970 (33.58)	150052 (31.26)
**Physical activity**			
Regular	1140 (64.41)	1940 (67.15)	333220 (69.42)
Unregular	582 (32.88)	873 (30.22)	135572 (28.25)
NA	48 (2.71)	76 (2.63)	11181 (2.33)
**Body mass index, n (%)**			
Underweight (<18.5)	24 (1.36)	9 (0.31)	2455 (0.51)
Normal weight (18.5-25)	551 (31.13)	886 (30.67)	155746 (32.45)
Overweight (25-30)	730 (41.24)	1261 (43.65)	203271 (42.35)
Obese (>30)	455 (25.71)	716 (24.78)	116374 (24.25)
NA	10 (0.56)	17 (0.59)	2127 (0.44)
**Proccessed meat, n (%)**			
Health	642 (36.27)	1072 (37.11)	190328 (39.65)
Unhealthy	1121 (63.33)	1803 (62.41)	287899 (59.98)
NA	7 (0.40)	14 (0.48)	1746 (0.37)
**Education level, n (%)**			
College	473 (26.72)	755 (26.13)	155692 (32.44)
Non-college	1271 (71.81)	2100 (72.69)	319058 (66.47)
NA	26 (1.47)	34 (1.18)	5223 (1.09)

Table S2. Effect estimates of two-sample MR analysis of smoking behaviors with IBD

Outcome	Exposure	Method	nsnp	b	se	pval	OR	LCI	UCI
CD	SmkAge	MR Egger	6	1.073	1.985	0.617	2.92	0.06	142.97
		Weighted median	6	-0.452	0.545	0.406	0.64	0.22	1.85
		Inverse variance weighted	6	-0.363	0.456	0.427	0.70	0.28	1.70
		Simple mode	6	-0.702	0.811	0.426	0.50	0.10	2.43
		Weighted mode	6	-0.652	0.832	0.468	0.52	0.10	2.66
CD	SmkCes	MR Egger	11	0.539	0.882	0.556	1.71	0.30	9.66
		Weighted median	11	0.105	0.231	0.650	1.11	0.71	1.75
		Inverse variance weighted	11	-0.174	0.296	0.557	0.84	0.47	1.50
		Simple mode	11	0.351	0.350	0.338	1.42	0.72	2.82
		Weighted mode	11	0.252	0.261	0.356	1.29	0.77	2.14
CD	SmkInit	MR Egger	267	-0.384	0.325	0.239	0.68	0.36	1.29
		Weighted median	267	0.076	0.092	0.408	1.08	0.90	1.29
		Inverse variance weighted	267	0.132	0.079	0.096	1.14	0.98	1.33
		Simple mode	267	0.076	0.293	0.795	1.08	0.61	1.91
		Weighted mode	267	0.076	0.247	0.759	1.08	0.66	1.75
CD	CigDay	MR Egger	35	-0.147	0.244	0.551	0.86	0.54	1.39
		Weighted median	35	0.039	0.163	0.810	1.04	0.76	1.43
		Inverse variance weighted	35	0.107	0.144	0.454	1.11	0.84	1.48
		Simple mode	35	0.419	0.378	0.276	1.52	0.72	3.19
		Weighted mode	35	0.052	0.167	0.758	1.05	0.76	1.46
UC	SmkAge	MR Egger	6	0.593	2.782	0.842	1.81	0.01	422.07
		Weighted median	6	0.353	0.605	0.559	1.42	0.43	4.66
		Inverse variance weighted	6	0.415	0.575	0.471	1.51	0.49	4.67
		Simple mode	6	0.531	0.930	0.593	1.70	0.27	10.52
		Weighted mode	6	0.445	0.913	0.647	1.56	0.26	9.34
UC	SmkCes	MR Egger	11	0.157	0.804	0.850	1.17	0.24	5.65
		Weighted median	11	-0.078	0.241	0.745	0.92	0.58	1.48
		Inverse variance weighted	11	-0.165	0.263	0.531	0.85	0.51	1.42
		Simple mode	11	0.028	0.384	0.943	1.03	0.48	2.18
		Weighted mode	11	0.042	0.265	0.878	1.04	0.62	1.75
UC	SmkInit	MR Egger	267	-0.190	0.304	0.533	0.83	0.46	1.50
		Weighted median	267	0.085	0.088	0.334	1.09	0.92	1.29
		Inverse variance weighted	267	-0.016	0.074	0.830	0.98	0.85	1.14
		Simple mode	267	0.169	0.297	0.571	1.18	0.66	2.12
		Weighted mode	267	0.191	0.243	0.432	1.21	0.75	1.95
UC	CigDay	MR Egger	34	-0.054	0.223	0.811	0.95	0.61	1.47
		Weighted median	34	-0.100	0.154	0.518	0.91	0.67	1.22
		Inverse variance weighted	34	-0.172	0.129	0.181	0.84	0.65	1.08
		Simple mode	34	-0.168	0.318	0.600	0.84	0.45	1.58
		Weighted mode	34	-0.073	0.148	0.624	0.93	0.70	1.24

agesmk, initiation of regular smoking. smkces, smoking cessation. smkinit, whether an individual had ever smoked regularly. cigday, the average number of cigarettes smoked per day. CD, Crohn's disease. UC, ulcerative colitis. nsnp, the number of SNPs used in the MR analysis. OR, odds ratio. LCI, low 95% confidence interval. UCI, upper 95% confidence interval.

Table S3. MP-PRESSO results of two-sample MR analysis of smoking behaviors with IBD

exposure	outcome	N_SNP	Outlier	Global test. P value	Distortion test. P value	MR analysis	Beta	SE	P Value
agesmk	CD	6	0	0.203	NA	Raw	-0.15	0.39	0.718
						Outlier-corrected	NA	NA	NA
agesmk	UC	6	0	0.065	NA	Raw	0.39	0.47	0.431
						Outlier-corrected	NA	NA	NA
smkces	CD	11	1	<0.001	0.197	Raw	-0.16	0.22	0.490
						Outlier-corrected	-0.04	0.19	0.825
smkces	UC	11	1	0.009	0.142	Raw	-0.15	0.20	0.459
						Outlier-corrected	-0.03	0.16	0.837
smkinit	CD	267	7	<0.001	0.909	Raw	0.13	0.07	0.092
						Outlier-corrected	0.13	0.07	0.049
smkinit	UC	267	3	<0.001	0.912	Raw	-0.02	0.07	0.797
						Outlier-corrected	-0.03	0.07	0.670
cigday	CD	35	2	<0.001	0.145	Raw	0.11	0.15	0.479
						Outlier-corrected	0.03	0.13	0.827
cigday	UC	34	0	0.027	NA	Raw	-0.13	0.14	0.350
						Outlier-corrected	NA	NA	NA

agesmk, initiation of regular smoking. smkces, smoking cessation. smkinit, whether an individual had ever smoked regularly. cigday, the average number of cigarettes smoked per day. CD, Crohn's disease. UC, ulcerative colitis. N_SNP, the number of SNPs used in the MR analysis.

Table S4. The demographic characteristics of the IBD-CHARACTER cohort. (Kalla et al. 2023. PMID: 36029471)

**Variables**	**IBD (N=343)**	**control (N=295)**
**Males [%]**	185 [54]	130 [44]
**Subtype IBD [CD:UC:IBD-U]**	154:161:28	
**Subtype controls [HC:non-IBD]**		52:241
**Smoking status [current:never:ex-smoker:missing]**	54:147:103:39	53:147:58:37
**Mean age, years [range]**	34 [7-79]	33 [3-79]
**Montreal classification of IBD**		
L1	48, 1 [31%, 1%]	
L2, +L4	41, 5 [27%, 3%]	
L3, +L4	46, 10 [30%, 6%]	
Isolated L4	3 [2%]	
**Montreal behaviour for CD**		
B1, B1p [non-stricturing and non-penetrating, +perianal]	120, 6 [78%, 4%]	
B2, B2p [stricturing, +perianal]	12, 0 [8%, 0%]	
B3, B3p [penetrating, +perianal]	6, 6 [4%, 4%]	
Not available	4 [2%]	
**Paris extent for UC**		
E1	41 [25%]	
E2	52 [32%]	
E3	67 [42%]	
Not available	1 [1%]	

IBD, inflammatory bowel disease. CD, Crohn’s disease. UC, ulcerative colitis. IBD-U, unspecific classified IBD.

Table S5. Colocalization analysis for CD and UC

IBD subtype	CpG	n_mQTL	mQTL	Gene	PP.H0	PP.H1	PP.H2	PP.H3	PP.H4
CD	cg03599224	2	rs1799964/rs2240064	*LTA*	6.19E-35	3.00E-28	1.05E-09	0.00	1.00
CD	cg17742416*	1	rs13428812	*DNMT3A*	/	/	/	/	/
CD	cg25607920	6	rs11210529/rs115798440/rs2985846/rs74067948/rs7534228/rs9661982	*HIVEP3*	6.63E-231	2.24E-232	9.53E-01	0.03	0.01
CD	cg03884592	8	rs11209970/rs11210529/rs11210543/rs115798440/rs687835/rs74067948/rs7534228/rs9661982		3.61E-276	1.31E-277	9.56E-01	0.03	0.01
CD	cg15937073	8	rs11210529/rs11210543/rs114909830/rs115798440/rs12048382/rs687835/rs74067948/rs9661982		1.61E-271	5.87E-273	7.84E-01	0.03	0.19
CD	cg26038582	8	rs11209970/rs11210529/rs11210543/rs11210628/rs687835/rs74067948/rs7518763/rs9661982		6.12E-241	2.05E-242	9.51E-01	0.03	0.02
CD	cg16145216	3	rs12124991/rs143577849/rs796724		2.58e-313	3.43e-315	8.64E-01	0.01	0.13
CD	cg04641860	2	rs1106215/rs12221341	*HNRNPF*	1.50E-14	3.87E-16	7.50E-01	0.02	0.23
CD	cg21920570	3	rs144911049/rs7926608/rs9943597	*MACROD1*	1.24E-72	9.97E-74	4.77E-01	0.04	0.49
UC	cg14647125	2	rs1035909/rs35342079	*AHRR*	3.54E-74	4.16E-71	8.26E-04	0.97	0.03
UC	cg23916896	2	rs2672761/rs4957023		1.81E-47	2.19E-48	7.99E-01	0.10	0.11
UC	cg17742416*	1	rs13428812	*DNMT3A*	/	/	/	/	/

* There were less than 10 available mQTLs for cg17742416, it therefore was excluded from the colocalization analysis.

n_mQTL, the number of mQTL for the CpG site used in epigenic MR analysis. CD, Crohn's disease. UC, ulcerative colitis.

In colocalization analysis, the posterior probability of five hypotheses were tested: i) H0, Neither trait in this region is genetically related (PP.H0); ii) H1, Only trait 1 has a genetic association in this region (PP.H1); iii) H2, Only trait 2 has a genetic association in this region (PP.H2); iv) H3, Both traits are correlated, but the causal variables are different (PP.H3); and v) H4, Both traits are correlated and share a causal variable (PP.H4).

Table S6. Definition of inflammatory bowel disease in the UK Biobank.

IBD definition	ICD Code
Crohn's disease	ICD 9: 555, 5550, 5551, 5552, 5559
	ICD 10: K500, K501, K508, K509
Ulcerative colitis	ICD 9: 556, 5569
	ICD 10: K510, K512, K513, K514, K515, K518, K519
