# Supplementary material for: Altered DNA methylation within DNMT3A, AHRR, LTA/TNF loci mediates the effect of smoking on inflammatory bowel disease
Source: Nat Commun. 2024 Jan 18;15:595. doi: 10.1038/s41467-024-44841-y (PMC10796384; doi:10.1038/s41467-024-44841-y)
Supplement: Supplementary file 3 — Description of Additional Supplementary Files [file 41467_2024_44841_MOESM3_ESM.pdf]

Description of Additional Supplementary Files

File Name: Supplementary Data 1
Description: MR results of smoking-related DNA methylation with CD and UC risk.

File Name: Supplementary Data 2
Description: Horizontal pleiotropy detected by MR-Egger method in the analysis of smoking-related DNA methylation with CD and UC risk.

File Name: Supplementary Data 3
Description: The differentially methylated genetic loci between CD patients and controls.

File Name: Supplementary Data 4
Description: The differentially methylated genetic loci between UC patients and controls.

File Name: Supplementary Data 5
Description: The differentially methylated genetic loci between CD patients and controls in whom smoking history was available, and excluding those for whom a smoking history was not taken.

File Name: Supplementary Data 6
Description: The differentially methylated genetic loci between UC patients and controls in whom smoking history was available, and excluding those for whom a smoking history was not taken.

File Name: Supplementary Data 7
Description: Prospective mQTL-smoking interaction in UK Biobank.

File Name: Supplementary Data 8
Description: The instrumental variables used in two-sample MR analysis of smoking behaviors with IBD risk.

File Name: Supplementary Data 9
Description: Statistically significant CpGs in relation to smoking status (Joehanes et al).
