# Supplementary material for: Altered DNA methylation within DNMT3A, AHRR, LTA/TNF loci mediates the effect of smoking on inflammatory bowel disease
Source: Nat Commun. 2024 Jan 18;15:595. doi: 10.1038/s41467-024-44841-y (PMC10796384; doi:10.1038/s41467-024-44841-y)
Supplement: Supplementary file 5 — Reporting Summary [file 41467_2024_44841_MOESM5_ESM.pdf]

nature portfolio

Corresponding author(s): Xue Li

Last updated by author(s): Dec 13, 2023

Reporting Summary

Nature Portfolio wishes to improve the reproducibility of the work that we publish. This form provides structure for consistency and transparency in reporting. For further information on Nature Portfolio policies, see our Editorial Policies and the Editorial Policy Checklist.

Statistics

For all statistical analyses, confirm that the following items are present in the figure legend, table legend, main text, or Methods section.

n/a	Confirmed	
☐	☒	The exact sample size (*n*) for each experimental group/condition, given as a discrete number and unit of measurement
☒	☐	A statement on whether measurements were taken from distinct samples or whether the same sample was measured repeatedly
☐	☒	The statistical test(s) used AND whether they are one- or two-sided<br>*Only common tests should be described solely by name; describe more complex techniques in the Methods section.*
☐	☒	A description of all covariates tested
☐	☒	A description of any assumptions or corrections, such as tests of normality and adjustment for multiple comparisons
☐	☒	A full description of the statistical parameters including central tendency (e.g. means) or other basic estimates (e.g. regression coefficient) AND variation (e.g. standard deviation) or associated estimates of uncertainty (e.g. confidence intervals)
☐	☒	For null hypothesis testing, the test statistic (e.g. *F*, *t*, *r*) with confidence intervals, effect sizes, degrees of freedom and *P* value noted<br>*Give P values as exact values whenever suitable.*
☒	☐	For Bayesian analysis, information on the choice of priors and Markov chain Monte Carlo settings
☐	☒	For hierarchical and complex designs, identification of the appropriate level for tests and full reporting of outcomes
☒	☐	Estimates of effect sizes (e.g. Cohen's *d*, Pearson's *r*), indicating how they were calculated

Our web collection on statistics for biologists contains articles on many of the points above.

Software and code

Policy information about availability of computer code

Data collection	The dataset of the UK Biobank were obtained through the application number 73595 and prepared by using R software (4.1.3). The data fro Mendelian randomization analysis and colocalization analysis were obtained from the published GWAS or EWAS studies and also prepared through R software (4.1.3) with the codes we have provided in the GitHub (https://github.com/XueLab157/Smoking-related-DNA-methylation-and-IBD#smoking-related-dna-methylation-and-ibd). For the genome-wide DNA methylation analysis, the detailed data collection has been described by Kalla Rahul. et al. in the Journal Crohn's and Colitis (PMID: 36029471. DOI: 10.1093/ecco-jcc/jjac127).
Data analysis	All software used is publicly available and described in the Methods section of this study. The codes for Cox regression, Mendelian randomization study, genome-wide DNA methylation analysis, and colocalization analysis are deposited in the Github (https://github.com/XueLab157/Smoking-related-DNA-methylation-and-IBD#smoking-related-dna-methylation-and-ibd)76. Mendelian randomization analyses were conducted using the TwoSampleMR package in R version 0.5.6 (https://mrcieu.github.io/TwoSampleMR/), genetic colocalization analyses were conducted using the coloc package (5.1.0.1) in R (https://github.com/chr1swallace/coloc) and visualized using the locuscomparer package (1.0.0) in R (https://github.com/boxiangliu/locuscompare, using default priors).

For manuscripts utilizing custom algorithms or software that are central to the research but not yet described in published literature, software must be made available to editors and reviewers. We strongly encourage code deposition in a community repository (e.g. GitHub). See the Nature Portfolio guidelines for submitting code & software for further information.

Data

Policy information about availability of data

All manuscripts must include a data availability statement. This statement should provide the following information, where applicable:

- Accession codes, unique identifiers, or web links for publicly available datasets
- A description of any restrictions on data availability
- For clinical datasets or third party data, please ensure that the statement adheres to our policy

The UK Biobank dataset can be accessed by researchers through a registration and application process at http://ukbiobank.ac.uk/registerapply/, as it serves as an open-access resource for scientific investigations. The UK Biobank application number of this study is 73595. The dataset generated from the UK Biobank to perform Cox regression and G-E interaction analysis are deposited as Source Data for Table 1, 2, and Supplementary Data 7 in the Figshare Repository (https://doi.org/10.6084/m9.figshare.24791634.v2). The GWAS data for tobacco smoking utilized in this study can be accessed via the GSCAN data portal (https://conservancy.umn.edu/handle/11299/201564). When using the dataset, authors should cite the original publication. The IBD summary statistics can be obtained from the GWAS Catalog under accession codes GCST004132 and GCST004133, respectively. The smoking-SNPs, and smoking-related CpGs used in this study can be found in Supplementary Data 8 and 9, respectively. The mQTLS used in epigenetic MR analysis and colocalization analysis were derived from the GoDMC consortium (http://mqtldb.godmc.org.uk/) by using the code which we have provided. The Source data for Figure 1, 2, and 3 in this study have also been deposited in the Figshare repository (https://doi.org/10.6084/m9.figshare.24791634.v2). The data used to perform genome-wide DNA methylation analysis cannot be made publicly available on account of data privacy laws and the national GDPR restrictions affecting some of the collaborating institutions within the IBD-Character Consortium. However, the data can be obtained from the corresponding author upon reasonable request (Jack Satsangi, E-mail: jack.satsangi@ndm.ox.ac.uk.) The request would be responded within one week. When using the data, authors should acknowledge the IBD-Character Consortium and its staff. Source data are provided with this paper. All software used is publicly available and described in the Methods section of this study. The codes for Cox regression, Mendelian randomization study, genome-wide DNA methylation analysis, and colocalization analysis are deposited in the Github (https://github.com/XueLab157/Smoking-related-DNA-methylation-and-IBD#smoking-related-dna-methylation-and-ibd)76. Mendelian randomization analyses were performed in R based on the 'TwoSampleMR' (version 0.5.6) package (https://mrcieu.github.io/TwoSampleMR/), genetic colocalization analyses were conducted through the 'coloc' (5.1.0.1) package (https://github.com/chr1swallace/coloc) and visualized using the 'locuscomparer' (1.0.0) package in R (https://github.com/boxiangliu/locuscompare, using default priors).

Research involving human participants, their data, or biological material

Policy information about studies with human participants or human data. See also policy information about sex, gender (identity/presentation), and sexual orientation and race, ethnicity and racism.

Reporting on sex and gender	The sex and gender were not considered in this study.
Reporting on race, ethnicity, or other socially relevant groupings	Our study mainly focused on the European population. We used multi variable model and Mendelian randomization design to control for confounding variables.
Population characteristics	The mean age for Crohn's disease cases, ulcerative colitis cases and controls were 56.78 (8.14), 57.6 (7.86), and 56.53 (8.09), respectively. The population characteristics of the participants of GWAS and EWAS study have been described in the original studies.
Recruitment	The UK Biobank. Relevant GWAS and EWAS studies. The IBD-CHARACTER inception cohort.
Ethics oversight	The UK Biobank obtained ethical approval from the North West-Haydock Research Ethics Committee (REC reference: 16/NW/0274). The genome-wide methylation association study, conducted on a subset of the IBD-CHARACTER inception cohort, was approved by the Regional Committee for Medical Research Ethics, South-Eastern Norway (REK sør-øst 2009/2015) and received endorsement from the privacy protection representative at Akershus University Hospital (13-123). The ethical approval of published GWASs and EWASs was obtained from their corresponding review board.

Note that full information on the approval of the study protocol must also be provided in the manuscript.

Field-specific reporting

Please select the one below that is the best fit for your research. If you are not sure, read the appropriate sections before making your selection.

☒ Life sciences ☐ Behavioural & social sciences ☐ Ecological, evolutionary & environmental sciences

For a reference copy of the document with all sections, see nature.com/documents/nr-reporting-summary-flat.pdf

Life sciences study design

All studies must disclose on these points even when the disclosure is negative.

Sample size	After applying data exclusion criteria, a total of 481,743 (1,770 incident CD cases) and 482,862 (2,889 incident UC cases) individuals were included in the prospective cohort study. For genome-wide DNA methylation analysis, IBD patients were recruited based on a thorough clinical, microbiological, endoscopic, histological, and radiological evaluatio. A total of 295 controls, 154 CD and 161 UC patients were included in this analysis. The sample size was chosen based on the data that were available when the study was launched since no prior sample size estimates had been made.

Data exclusions	In the UK Biobank, participants without complete information on smoking behaviors, covariates, and genetic information, or with IBD diagnosis before baseline, or unspecific IBD diagnosis were excluded from the dataset. In Mendelian randomization analysis, SNPs or mQTLs with LD were excluded. In the genome-wide methylation analysis, those samples and probes which did not meet the criteria for analysis were removed.
Replication	The results of prospective cohort study was replicated in this study through a age-stratified analysis, and the results of replication have been described in the manuscript. For the epigenetic Mendelian randomization results which passed the multiple comparisons correction, we replicated the significant signal in an independent genome-wide DNA methylation analysis, the results were also reported in the manuscript.
Randomization	This is not relevant to this study because it is a population-based study.
Blinding	This is not relevant to this study because it is an observational study.

Reporting for specific materials, systems and methods

We require information from authors about some types of materials, experimental systems and methods used in many studies. Here, indicate whether each material, system or method listed is relevant to your study. If you are not sure if a list item applies to your research, read the appropriate section before selecting a response.

Materials & experimental systems

n/a	Involved in the study
☒	☐ Antibodies
☒	☐ Eukaryotic cell lines
☒	☐ Palaeontology and archaeology
☒	☐ Animals and other organisms
☒	☐ Clinical data
☒	☐ Dual use research of concern
☒	☐ Plants

Methods

n/a	Involved in the study
☒	☐ ChIP-seq
☒	☐ Flow cytometry
☒	☐ MRI-based neuroimaging
